# Effects of self-regulated learning prompts on statistical problem-solving by goal orientation

**DOI:** 10.3389/fpsyg.2026.1967032

**Published:** 2026-10-29

**Authors:** Aeng Muhidin, Shinta Doriza

**Affiliations:** 1 Program Studi Pendidikan Pancasila dan Kewarganegaraan, Universitas Pamulang, Jakarta, Indonesia; 2 Program Studi Pendidikan Kesejahteraan Keluarga, Universitas Negeri Jakarta, Jakarta, Indonesia

**Keywords:** achievement goal orientation, aptitude-treatment interaction, metacognitive prompt, self-regulated learning, statistical problem-solving

## Abstract

Complex statistical problem-solving requires students to plan, monitor, and evaluate their own thinking, yet instructional interventions designed to support this self-regulation often produce inconsistent results. This study examined whether a metacognitive-prompt intervention grounded in self-regulated learning (SRL) theory was associated with university students’ statistical problem-solving ability, and whether this association depended on students’ achievement goal orientation (mastery vs. performance). A quasi-experimental, nonequivalent control-group pretest-posttest design with a 2 × 2 factorial structure (class × orientation) was used with 21 students from two intact classes (experimental *n* = 10, control *n* = 11); because class was assigned at the classroom rather than the individual level, the study is best characterized as a small, single-site pilot comparison. Achievement goal orientation was measured with an instrument adapted from a 2 × 2 achievement goal framework, and statistical problem-solving was measured with a 10-item complex multiple-choice test. Two-way ANOVA on normalized gain (N-gain) scores, supported by ANCOVA controlling for pretest, showed no statistically significant main effect of the metacognitive-prompt intervention, *F* (1,17) = 0.02, *p* = 0.882, partial η^2^ = 0.001, 95% CI [0.00, 0.46]; no significant main effect of achievement goal orientation, *F* (1,17) = 1.59, *p* = 0.224, partial η^2^ = 0.086, 95% CI [0.00, 0.54]; and no significant interaction between the two, *F* (1,17) = 0.08, *p* = 0.779, partial η^2^ = 0.005, 95% CI [0.00, 0.47]. A sensitivity analysis showed the design had 80% power to detect only very large effects (partial η^2^ ≥ 0.30), and equivalence tests did not support concluding that the true effects were negligible. All four groups achieved a moderate mean N-gain (0.30–0.70). These findings do not support the hypothesized aptitude-treatment interaction; rather than showing that prompting has no effect, this exploratory, underpowered comparison detected no statistically significant differential benefit across goal orientations, and a larger, multi-classroom design is needed for a confirmatory test.

## 1 Introduction

Statistical problem-solving that goes beyond routine computation to include selecting appropriate procedures, checking assumptions, and interpreting results in context is a persistent difficulty for university students taking introductory statistics courses. Unlike single-step calculation, this type of complex problem-solving requires students to actively plan how to approach a task, monitor their reasoning while working through it, and evaluate whether their conclusions are justified — the core cyclical phases of self-regulated learning (SRL) described by Zimmerman (2002). Students who regulate their own thinking during a task tend to select more appropriate strategies and catch their own errors, whereas students who apply procedures without reflection are more likely to misuse formulas or misinterpret statistical output. Self-regulated learning is cyclical, moving through a forethought phase (goal setting and strategic planning), a performance phase (self-monitoring and strategy use), and a self-reflection phase (self-evaluation and adaptation), each of which feeds into the next attempt at a task (Panadero, 2017).

One common way to support SRL during a learning task is through metacognitive prompts, as brief cues inserted into instruction that ask students to plan, monitor, or evaluate their own thinking as they work (Azevedo and Cromley, 2004). Azevedo and Cromley (2004) found that students who received such prompts while learning with a hypermedia environment developed more accurate mental models than students who worked without prompting, indicating that externally triggered self-regulation can substitute, at least temporarily, for self-regulation that a student has not yet internalized. In domains such as mathematics, prompting students to consider what a problem is asking, which strategy applies, and whether an answer makes sense has long been associated with better problem-solving performance (Garofalo and Lester, 1985; Schoenfeld, 2016), and reviews of metacognitive instruction report generally positive, though not universal, effects on learning outcomes (Veenman et al., 2006). More recent work has continued to refine this picture. Zeitlhofer et al. (2023) found that the effectiveness of cognitive and metacognitive prompts depended on students’ prior knowledge and strategy use rather than operating uniformly. Wang et al. (2023) showed that computer-based scaffolds shaped students’ metacognitive monitoring and problem-solving efficiency in an intelligent tutoring system, and Shin et al. (2023) found that combining worked-out examples with metacognitive scaffolding improved programming problem-solving beyond either component alone. Even newer generative-AI-based tutoring environments depend on similar metacognitive support to translate their potential into actual self-regulated learning gains (X. Xu et al., 2025). Recent meta-analyses reinforce this pattern: self-regulated-learning interventions show a moderate positive average effect on academic achievement in online and blended settings (Guntur and Purnomo, 2024; Z. Xu et al., 2023), a pattern echoed in second-language learning contexts as well (Chen, 2022), and scaffolding more broadly produces a large effect on problem-solving ability (Gou et al., 2025), yet the same syntheses report considerable heterogeneity across studies, indicating that prompting and scaffolding do not benefit every learner or every task to the same degree. These findings suggest that a similar prompting approach could be adapted to support statistical problem-solving, which shares with mathematical problem-solving a reliance on procedural judgment, assumption-checking, and interpretation. Complex problem-solving in mathematics and statistics differs from routine computation in that it requires a solver to orient to the problem, plan and organize a solution path, execute the chosen strategy, and verify the result against the original context — the four metacognitive categories identified by Garofalo and Lester (1985). Recent studies in statistics education support this emphasis on metacognitive monitoring. Makwakwa et al. (2024) found that first-year undergraduates’ statistical problem-solving skills were weakest precisely at the verification stage, and technology-enhanced, metacognitively scaffolded instruction has shown promise in this domain (Hidayat et al., 2025; Tan et al., 2023a; Tan et al., 2023b). However, the achievement goal literature indicates that instructional supports are not uniformly effective for all learners. Dweck (1986) showed that students holding a mastery goal, who define success as developing their own competence, tend to respond to a task with effort and self-regulatory strategies, while students holding a performance goal, who define success as demonstrating competence relative to others, are more likely to rely on shortcuts or to disengage when a task becomes difficult. Elliot and McGregor (2001) formalized this distinction in a 2 × 2 achievement goal framework that separates mastery and performance goals along an approach-avoidance dimension, and demonstrated that the two orientations relate to distinct patterns of study strategies, test anxiety, and performance. Contemporary research continues to confirm that mastery- and performance-approach goals lead to distinct patterns of effort, progress, and affect during goal pursuit (Katz-Vago and Benita, 2024), that performance-avoidance goals are associated with anxiety and depressive symptoms (Diaconu-Gherasim et al., 2024), and that the trajectories of the four 2 × 2 goals across a college career predict later academic performance (Liu et al., 2023). Building on Snow's (1989) aptitude-treatment interaction (ATI) framework, in which the effectiveness of an instructional treatment depends on a learner characteristic, it is plausible that metacognitive prompts benefit mastery-oriented students — whose goal is already aligned with self-monitoring and understanding — more than performance-oriented students, who may treat the prompts as an additional demand rather than a resource. This aptitude-treatment logic continues to find direct support in current classroom research; for example, Hofer and Reinhold (2025) tested several instructional scaffolds for teaching fractions and found no universal effect of any single scaffold, with different scaffolds instead benefiting students with different ability and interest profiles. Despite this theoretical rationale, few studies have directly tested whether the effect of a metacognitive-prompt intervention on complex statistical problem-solving differs across achievement goal orientations; most available evidence addresses either metacognitive prompts or achievement goals in isolation. This gap is the empirical novelty this study addresses.

This study therefore asks whether the effect of a metacognitive-prompt intervention on university students’ statistical problem-solving ability depends on their achievement goal orientation. It was hypothesized that an interaction would be found, such that mastery-oriented students in the experimental (prompted) class would show larger gains than performance-oriented students, while orientation would matter less in the control (unprompted) class. Accordingly, this study aimed to examine (a) the main effect of a metacognitive-prompt intervention, (b) the main effect of achievement goal orientation, and (c) the interaction between the two, on students’ statistical problem-solving ability, assessed through pretest–posttest gain scores.

## 2 Materials and methods

This study used a quasi-experimental, nonequivalent control-group pretest-posttest design with a 2 × 2 factorial structure crossing class (experimental vs. control) with achievement goal orientation (mastery vs. performance). Because the two classes were pre-existing (intact) groups rather than the result of individual random assignment, the design followed the logic of aptitude-treatment interaction research (Snow, 1989), in which a learner characteristic assessed prior to treatment is used to test whether an instructional treatment interacts with that characteristic.

Participants were 21 university students enrolled in a statistics course at Universitas Pamulang, drawn from two intact classes. The experimental class (*n* = 10) consisted of 5 students classified as mastery-oriented and 5 classified as performance-oriented; the control class (*n* = 11) consisted of 6 mastery-oriented and 5 performance-oriented students, based on their achievement goal orientation scores collected before treatment.

Two instruments were used. Achievement goal orientation was measured with an instrument adapted from Elliot and McGregor’s (2001) 2 × 2 achievement goal framework, which assesses mastery-approach, mastery-avoidance, performance-approach, and performance-avoidance goals (the original instrument comprises 12 items, three per subscale, rated on a 1–7 scale, with subscale reliabilities of *α* = 0.83–0.92 in Elliot and McGregor's, 2001 validation sample). For use in the present study, the instrument was translated and linguistically adapted into Indonesian while retaining the conceptual meaning of the original items. The adaptation focused primarily on contextual and linguistic appropriateness so that the statements could be readily understood by the participants without changing the theoretical meaning of the constructs. The modifications involved replacing context-specific expressions with wording that was more appropriate for the learning context of the participants. Examples of the modified items are presented to illustrate the adaptation process. For the mastery-approach dimension, the item was contextualized to emphasize the student’s effort to develop competence and gain deeper understanding, for example: “I strive to understand the learning material more deeply so that my abilities continue to develop.” Another possible formulation within this dimension emphasizes improvement in one’s own competence: “I am striving to improve my ability to understand the material being studied.” For the mastery-avoidance dimension, the wording was adapted to emphasize avoiding inadequate understanding or failing to master the material, for example: “I strive not to fail to grasp the material I am supposed to master.” A further example is: “I strive to ensure there is no important material that I do not understand.” For the performance-approach dimension, the wording was adapted to emphasize demonstrating superior competence relative to other students, for example: “I try to get better learning results compared to my friends.” Another example is: “Saya berusaha menunjukkan bahwa kemampuan belajar saya lebih baik daripada teman-teman saya.” These examples illustrate the contextual modification of the instrument. The modifications were intended to make the items appropriate to the educational and cultural context of the present study, while preserving the theoretical distinction among mastery-approach, mastery-avoidance, performance-approach, and performance-avoidance orientations. The adaptation did not alter the underlying constructs or their intended direction. The adapted instrument was reviewed for linguistic clarity, contextual appropriateness, and conceptual equivalence before administration.

For classification of students’ achievement-goal orientation, the scores of the relevant subscales were calculated by summing the item scores within each dimension. Mastery-oriented achievement goals were represented by the combined mastery-approach and mastery-avoidance scores, whereas performance-oriented achievement goals were represented by the combined performance-approach and performance-avoidance scores. The orientation with the higher summed score was identified as the student’s predominant achievement-goal orientation. If a tie occurred between the summed mastery and performance scores, the student was classified as having a mixed achievement-goal orientation, rather than being assigned arbitrarily to either category. If no tied scores occurred in the actual dataset, this rule was not invoked in the final classification.

For analysis, each student’s four subscale scores were used to classify the student into a single dominant orientation — mastery or performance — corresponding to whichever pair of subscales (mastery-approach/avoidance or performance-approach/avoidance) scored higher. The internal-consistency reliability of the adapted instrument was not assessed in the present sample, which we now note explicitly as a limitation (see Discussion). Statistical problem-solving ability was measured with a 10-item complex multiple-choice test covering measures of central tendency and variability, z-scores, sampling strategy, correlation, regression interpretation, hypothesis testing, and integrated statistical decision-making. Each item presented several statements, of which more than one could be correct, and was scored dichotomously — one point if a student selected exactly the correct combination of statements and zero otherwise — for a maximum possible score of 10. This complex multiple-choice format follows recently validated practice for assessing higher-order, verification-dependent reasoning rather than single-fact recall (Bhakti et al., 2025). The same test was administered as pretest and posttest.

Prior to treatment, all students completed the achievement goal questionnaire and the pretest. The experimental class then received a series of statistical problem-solving exercises embedded with metacognitive prompts — brief written cues before, during, and after each item (e.g., “What is this problem asking?,” “Which statistical concept applies here?,” “Does this answer make sense given the data?”) designed to trigger planning, monitoring, and evaluation. The control class completed the same set of statistical problem-solving exercises without these prompts. Both classes were taught by the same instructor using the same statistical content and the same amount of instructional time; the metacognitive prompts were the only feature that differed between conditions. After the treatment period, both classes completed the posttest.

An intervention involving metacognitive guidance was implemented during statistical problem-solving sessions in the experimental class, while the control class received standard instruction. The treatment period spanned two learning sessions, each lasting approximately 120 min. Throughout the intervention, students in the experimental class were systematically provided with metacognitive guidance designed to encourage them to plan their problem-solving approaches, monitor their understanding and progress, and evaluate the accuracy of their solutions. This guidance was integrated into the statistical problem-solving activities rather than being presented as a separate instructional topic. The control class received instruction covering the same statistical problem-solving material and was allotted roughly the same amount of instructional time as the experimental class, but without the structured metacognitive guidance intervention. Both classes were taught within the same institutional setting and followed the same relevant curriculum content and learning objectives. A post-test was administered 1 week after the final learning session. The same assessment framework for statistical problem-solving was used for both the pre-test and post-test, with procedures applied consistently across both classes.

To minimize potential contamination between the experimental and control conditions, several procedural measures were implemented. The two classes were scheduled at different times, and students were instructed not to disseminate or share intervention materials, metacognitive guidance, or related worksheets with students in the other class during the treatment period. Instructors also utilized distinct learning materials for each condition. These procedures were intended to reduce the likelihood of students in the control group being indirectly exposed to the metacognitive guidance intervention. Given that this study involved two pre-existing classes and class assignment was conducted at the group level rather than the individual level, these procedures were implemented primarily to maintain the distinction between the experimental and control conditions, rather than to claim the absolute prevention of contamination.

Pretest and posttest scores were used to compute a raw gain score (posttest − pretest) and a normalized gain (N-gain) score for each student, calculated as (posttest − pretest) / (maximum possible score − pretest), following Hake (1998); N-gain scores were categorized as high (≥ 0.70), moderate (0.30–0.69), or low (< 0.30). Shapiro–Wilk tests were used to check the normality of N-gain scores within each of the four class-by-orientation cells, and Levene’s test was used to check homogeneity of variance across cells. The main hypotheses were tested with a two-way analysis of variance (ANOVA) on N-gain scores, with class and achievement goal orientation as between-subjects factors, using Type III sums of squares to accommodate the unequal cell sizes. As a robustness check, an analysis of covariance (ANCOVA) was also conducted on posttest scores, with pretest scores entered as a covariate and class and orientation as factors; the homogeneity-of-regression-slopes assumption underlying this ANCOVA was tested directly with a three-way pretest × class × orientation interaction term. To quantify effect size, partial eta-squared was computed for every ANOVA and ANCOVA effect, together with 95% confidence intervals from a 5,000-replicate stratified (within-cell) bootstrap. Because Levene’s test indicated heterogeneity of variance (see Results), a Welch’s ANOVA and a 10,000-permutation randomization test were conducted as distribution-free robustness checks on the two-way ANOVA. Two one-sided tests (TOST) were used to test statistical equivalence against a smallest-effect-size-of-interest of Cohen’s d = 0.50, and a sensitivity power analysis was conducted to determine the minimum effect size this design could detect with 80% power at *α* = 0.05. All analyses were conducted in Python 3.10 using SciPy 1.15, statsmodels 0.14, and Pingouin 0.6, with *α* = 0.05; the full analysis code and package versions are provided as Supplementary Material.

## 3 Results

Table 1 presents descriptive statistics for pretest, posttest, raw gain, and N-gain scores in each of the four class-by-orientation cells.

**Table 1:** Descriptive statistics (M, SD) for pretest, posttest, raw gain, and normalized gain (N-gain) scores by class and achievement goal orientation.

Class	Orientation	*n*	Pretest M (SD)	Posttest M (SD)	Raw gain M (SD)	N-gain M (SD)
Control	Mastery	6	4.00 (1.67)	7.50 (0.84)	3.50 (1.64)	0.57 (0.13)
Control	Performance	5	3.80 (1.30)	6.20 (1.48)	2.40 (0.55)	0.40 (0.13)
Experimental	Mastery	5	6.20 (1.92)	8.20 (1.30)	2.00 (0.71)	0.55 (0.11)
Experimental	Performance	5	5.20 (2.17)	7.80 (1.79)	2.60 (2.79)	0.45 (0.45)

Maximum possible score on pretest/posttest = 10. N-gain = normalized gain (Hake, 1998).

As shown in Table 1, students in the experimental class began with a higher mean pretest score (*M* = 5.70, SD = 2.00) than students in the control class (*M* = 3.91, SD = 1.45); an analysis of variance on pretest scores confirmed this baseline difference was statistically significant, *F* (1, 17) = 5.40, *p* = 0.033, indicating that the two intact classes were not fully equivalent before treatment. All four class-by-orientation cells nevertheless reached a comparable, moderate mean N-gain (range 0.40–0.57, category “moderate” per Hake, 1998), suggesting a similar degree of relative improvement once the difference in starting scores is accounted for.

Shapiro–Wilk tests indicated that N-gain scores did not depart significantly from normality in any of the four cells (all *p* > 0.05). Levene’s test, however, indicated a significant violation of the homogeneity-of-variance assumption across the four cells, *F* (3, 17) = 4.66, *p* = 0.015, driven primarily by the larger variability of gain scores in the experimental-performance cell (SD = 0.45) compared to the other three cells (SD = 0.11–0.13). This heterogeneity, combined with the small and unequal cell sizes (n = 5–6), is taken into account when interpreting the ANOVA results below.

As shown in Table 2, the two-way ANOVA on N-gain scores revealed no statistically significant main effect of class, *F* (1, 17) = 0.02, *p* = 0.882, partial η^2^ = 0.001, 95% CI [0.00, 0.46]; no statistically significant main effect of achievement goal orientation, *F* (1, 17) = 1.59, *p* = 0.224, partial η^2^ = 0.086, 95% CI [0.00, 0.54]; and no statistically significant class-by-orientation interaction, *F* (1, 17) = 0.08, *p* = 0.779, partial η^2^ = 0.005, 95% CI [0.00, 0.47]. All three bootstrap confidence intervals were wide and spanned conventionally small-to-large values, reflecting the limited precision of an *n* = 21 design rather than evidence that the true effects are zero. An ANCOVA on posttest scores controlling for pretest produced a similar overall pattern: neither the main effect of class, *F* (1, 16) = 0.71, *p* = 0.411, partial η^2^ = 0.043, nor the main effect of orientation, *F* (1, 16) = 1.48, *p* = 0.242, partial η^2^ = 0.085, nor their interaction, *F*(1, 16) = 1.14, *p* = 0.301, partial η^2^ = 0.067, reached significance, while the pretest covariate approached but did not reach significance, *F* (1, 16) = 4.32, *p* = 0.054, partial η^2^ = 0.212. Because ANCOVA assumes homogeneous pretest-posttest regression slopes across cells, this assumption was tested directly: the three-way pretest × class × orientation interaction was statistically significant, *F* (1, 13) = 5.38, *p* = 0.037, indicating that the slope-homogeneity assumption was violated and that the ANCOVA results should be interpreted with caution rather than treated as a confirmatory robustness check. Table 3 reports two further robustness analyses conducted in light of the Levene’s-test violation reported above: a Welch’s ANOVA and a 10,000-permutation randomization test, both of which reproduced the same nonsignificant pattern as the parametric ANOVA, suggesting the null result is not an artifact of unequal variances. Table 3 also reports equivalence tests (TOST) against a smallest-effect-size-of-interest of Cohen’s d = 0.50: neither the class comparison, *p* = 0.160, nor the orientation comparison, *p* = 0.562, allowed statistical equivalence to be concluded, meaning the present data can neither confirm nor rule out a true effect of this magnitude. A sensitivity power analysis indicated that, with *n* = 21, statistical power of 80% was available only to detect effects of partial η^2^ ≥ 0.296 (equivalent to a two-group Cohen’s d ≈ 1.30); smaller, more typical educational-intervention effect sizes could not have been reliably detected in this design. Figure 1 displays the individual N-gain scores together with the mean and 95% confidence interval of each group; visually, the confidence intervals overlap substantially and, for the experimental-performance cell in particular, are very wide, consistent with the nonsignificant and imprecisely estimated statistical tests reported above.

**Table 2:** Two-way ANOVA on N-gain scores and ANCOVA on posttest scores (covariate: pretest), type III sums of squares, with partial eta-squared and 95% bootstrap confidence intervals.

Effect	SS	df	F	p	partial η^2^	95% CI
ANOVA on N-gain						
Class	0.001	1, 17	0.02	0.882	0.001	[0.00, 0.46]
Orientation	0.094	1, 17	1.59	0.224	0.086	[0.00, 0.54]
Class × Orientation	0.005	1, 17	0.08	0.779	0.005	[0.00, 0.47]
Residual	1.002	17	—	—	—	—
ANCOVA on posttest (covariate: pretest)						
Pretest	6.776	1, 16	4.32	0.054	0.212	—
Class	1.117	1, 16	0.71	0.411	0.043	—
Orientation	2.321	1, 16	1.48	0.242	0.085	—
Class × Orientation	1.796	1, 16	1.14	0.301	0.067	—
Residual	25.124	16	—	—	—	—
Homogeneity-of-regression-slopes check						
Pretest × Class × Orientation	7.243	1, 13	5.38	0.037	—	—

SS = Type III sum of squares. Bootstrap confidence intervals for partial η^2^ are based on 5,000 stratified (within-cell) resamples; intervals are not reported for the ANCOVA or the slope-homogeneity check because the latter indicates the ANCOVA’s equal-slopes assumption is violated (see Results and Discussion). — = not applicable.

**Table 3:** Robustness and sensitivity analyses conducted in light of the Levene’s-test violation and the small sample size.

Analysis	Statistic	df	*p*	Interpretation
Welch’s ANOVA – Class	*F* = 0.01	1, 12.7	0.929	Consistent with parametric ANOVA (null)
Welch’s ANOVA – Orientation	*F* = 1.63	1, 11.3	0.228	Consistent with parametric ANOVA (null)
Permutation ANOVA – Class	*F* = 0.02	—	0.883	Consistent with parametric ANOVA (null)
Permutation ANOVA – Orientation	*F* = 1.59	—	0.222	Consistent with parametric ANOVA (null)
Permutation ANOVA – Interaction	*F* = 0.08	—	0.780	Consistent with parametric ANOVA (null)
TOST equivalence – Class	bound = ±0.117 N-gain pts	—	0.160	Equivalence not established
TOST equivalence – Orientation	bound = ±0.117 N-gain pts	—	0.562	Equivalence not established
Sensitivity power analysis	minimum detectable *f* = 0.649 (partial η^2^ = 0.296, d ≈ 1.30)	1, 17	—	80% power only for very large effects

Welch’s ANOVA and the permutation test (10,000 permutations) are robustness checks for the two-way ANOVA on N-gain under variance heterogeneity. TOST = two one-sided tests, equivalence bound set to Cohen’s d = 0.50 (0.117 raw N-gain points, based on the pooled SD of 0.235). Sensitivity power analysis assumes *α* = 0.05, power = 0.80, dfₘₐₓₘ = 1, dfₑᵣᵣₒᵣ = 17.

**Figure 1:**
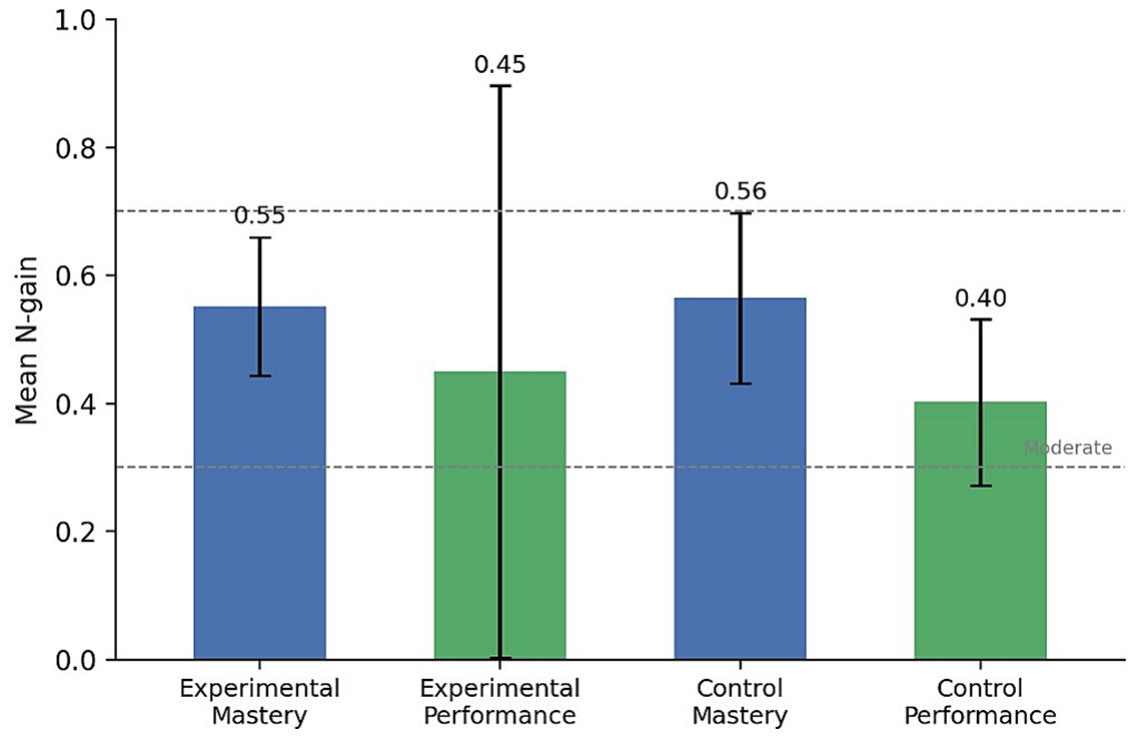
Individual normalized gain (N-gain) scores and group mean ± 95% confidence interval, by class and achievement goal orientation. Diamonds show the cell mean with its 95% CI; circles and squares show individual students’ N-gain scores.

Figure 1 shows that the control-mastery cell obtained the numerically highest mean N-gain [*M* = 0.57, 95% CI (0.43, 0.70)], followed closely by the experimental-mastery cell [*M* = 0.55, 95% CI [0.42, 0.69]), while both performance-oriented cells were numerically lower (experimental *M* = 0.45, 95% CI (−0.11, 1.01); control *M* = 0.40, 95% CI (0.24, 0.56)]. As a purely exploratory, hypothesis-generating comparison that was not part of the pre-specified analysis plan and was not corrected for multiple testing, an independent-samples t-test comparing mastery- and performance-oriented students within the control class yielded t(9) = 2.05, *p* = 0.071, Cohen’s d = 1.24; this nominally nonsignificant, uncorrected result should be treated as a descriptive observation to motivate future, adequately powered research rather than as evidence of a real orientation-related difference.

## 4 Discussion

### 4.1 The metacognitive prompt intervention did not produce a detectable main effect

Contrary to prior evidence that metacognitive prompting improves learning outcomes (Azevedo and Cromley, 2004), the experimental class did not show significantly larger gains than the control class in this study. Several features of the present design may explain this null result rather than contradicting the broader literature. First, the intervention in Azevedo and Cromley (2004) and in most published metacognitive-prompt studies was embedded across multiple extended sessions in a computer-based environment that logged and adapted to each learner’s behavior; the present intervention was comparatively brief and delivered through written cues rather than an adaptive system, which may have limited its dosage. Second, with only 21 students split across four cells, statistical power to detect a small-to-moderate effect was limited, so a true but modest benefit of prompting could easily have gone undetected — the sensitivity analysis reported in the Results indicates this design could only reliably detect effects of partial η^2^ ≥ 0.30. Third, several students in both classes posted high pretest scores and near-ceiling posttest scores (a maximum score of 10 was reached by some students in the experimental class), which compresses the range of possible gain and can attenuate an otherwise real treatment effect. This pattern is consistent with recent meta-analytic evidence that self-regulatory interventions in general — including self- and peer-assessment (Yan et al., 2022) and scaffolding more broadly (Gou et al., 2025) — tend to show positive but highly variable effects, with dosage, duration, and design quality acting as recurring moderators rather than exceptions.

### 4.2 Achievement goal orientation did not moderate the effect of the intervention

The hypothesized interaction between class and achievement goal orientation, motivated by Snow’s (1989) aptitude-treatment interaction framework and by theoretical expectations from Dweck (1986) and Elliot and McGregor (2001), was not supported. One possible explanation is measurement: the present study collapsed Elliot and McGregor’s (2001) four-category framework (mastery-approach, mastery-avoidance, performance-approach, performance-avoidance) into a simpler mastery-performance dichotomy, which may have obscured differences that are specific to the approach-avoidance dimension — for example, mastery-avoidance and performance-avoidance goals share an avoidance orientation that could respond differently to prompting than the approach-oriented forms of the same goals. A second explanation is that a single brief intervention may simply be an insufficient test of a learner-characteristic interaction that likely develops and manifests over a longer period of goal-directed engagement. It is also possible that goal orientation interacts less with a specific instructional prompt than with broader self-regulatory tendencies: academic self-regulation has been linked to lower procrastination and stress in secondary and college students (Elizondo et al., 2024; Ragusa et al., 2023), and achievement emotions such as anxiety and enjoyment have been shown to shape learning performance largely independently of the specific instructional format used (Qi et al., 2025); such broader dispositions were not measured here and may have masked a true orientation-by-prompt interaction.

### 4.3 Baseline non-equivalence and small cell sizes limit causal inference

Because the experimental and control classes were intact groups rather than randomly assigned individuals, the significant pretest difference between classes, *F* (1, 17) = 5.40, *p* = 0.033, is a threat to internal validity that is common in nonequivalent-groups quasi-experiments. The use of N-gain scores and an ANCOVA controlling for pretest was intended to partly address this imbalance, and both approaches converged on the same nonsignificant pattern, which strengthens confidence that the null result is not simply an artifact of the baseline difference. Even so, the violation of the homogeneity-of-variance assumption, *F* (3, 17) = 4.66, *p* = 0.015, together with cell sizes of only 5 to 6 students, means the present tests had limited power and should be interpreted as a preliminary rather than definitive test of the hypothesized interaction.

### 4.4 A descriptive pattern consistent with, but not confirming, the aptitude-treatment hypothesis

Although no comparison reached statistical significance, the numerically highest N-gain occurred among mastery-oriented students in the control class, who received no metacognitive prompting at all, and the marginal difference between mastery- and performance-oriented students within that class, *t* (9) = 2.05, *p* = 0.071, is worth noting rather than dismissing. This pattern is broadly consistent with theoretical descriptions of mastery orientation as intrinsically self-regulatory (Dweck, 1986; Zimmerman, 2002): students who are already inclined to plan, monitor, and evaluate their own learning may not need external prompts to do so, which would predict exactly the kind of ceiling on the added value of prompting observed here for mastery-oriented students. Because this pattern is descriptive rather than the result of a significant interaction test, it should be treated as a hypothesis for future, adequately powered research rather than as a confirmed finding.

### 4.5 Statistical power, instrument reliability, and the unit-of-analysis problem

Beyond the baseline non-equivalence discussed above, three further limitations qualify how the present results should be interpreted. First, because the experimental and control conditions were assigned at the level of intact classrooms rather than to individual students, observations within each class are not fully statistically independent: students in the same classroom shared the same instructor, instructional pacing, and classroom environment, which can make student-level significance tests appear more precise than the design actually supports (Theobald, 2018). With only two classrooms in the design, this non-independence cannot be modeled with a multilevel or cluster-robust analysis, because a random class effect is not statistically identifiable from a single classroom per condition; it can only be acknowledged as a structural limitation of a single-site, two-classroom comparison in which class is completely confounded with treatment. This is the central reason the present study is best framed as a descriptive, hypothesis-generating pilot comparison rather than a confirmatory test of the aptitude-treatment interaction hypothesis; a confirmatory test of this hypothesis will require multiple classrooms randomly assigned to each condition, so that classroom-level variability can be estimated and separated from student-level variability. Second, the internal-consistency reliability of the achievement-goal instrument and the statistical problem-solving test was not assessed in this sample; without a sample-specific reliability estimate, measurement error cannot be ruled out as a contributor to the null results, and future work should report Cronbach’s alpha or an equivalent reliability coefficient for both instruments. Third, as reported in the Results, a sensitivity power analysis indicated that the present design (*n* = 21) had 80% power to detect only very large effects (partial η^2^ ≥ 0.30, equivalent to a two-group Cohen’s d of approximately 1.30); consistent with this, equivalence testing could not confirm that the true effects were negligible either. Taken together, these three limitations mean the nonsignificant results reported here should not be read as evidence that metacognitive prompting has no effect for students of a particular achievement goal orientation, but as the outcome of an underpowered, single-site test that could not adequately distinguish a true null effect from a true but undetected one.

In conclusion, this study examined whether a metacognitive-prompt intervention grounded in self-regulated learning theory was associated with university students’ statistical problem-solving ability, and whether this association depended on students’ achievement goal orientation. Using a 2 × 2 quasi-experimental design with 21 students from two intact classrooms, two-way ANOVA on N-gain scores and a supporting ANCOVA both indicated no statistically significant main effect of the intervention, no statistically significant main effect of achievement goal orientation, and no statistically significant interaction between the two; equivalence tests further indicated that these null results cannot be interpreted as confirming negligible true effects. All four class-by-orientation groups achieved a moderate normalized gain, consistent with all groups improving over the course of the study; because this design lacks a no-instruction comparison group and cannot separate classroom-level from student-level variability, however, these gains cannot be attributed specifically to the metacognitive-prompt material itself, and the hypothesized aptitude-treatment interaction could not be confirmed or ruled out in this small, single-site sample. The findings are best read as a descriptive, hypothesis-generating pilot comparison rather than a confirmatory test.

Future research should replicate this design with a larger number of classrooms randomly assigned to condition, rather than a single classroom per condition, both to increase statistical power and to allow classroom-level variability to be modeled separately from student-level variability; retain Elliot and McGregor’s (2001) full four-category achievement goal classification rather than a collapsed mastery-performance dichotomy, to test whether approach and avoidance forms of each goal respond differently to prompting; report the internal-consistency reliability of all instruments in the sample in which they are used; extend the duration and intensity of the metacognitive-prompt intervention beyond a single brief exercise set; and consider process measures, such as think-aloud protocols or response-time data, to capture students’ self-regulation more directly than pretest-posttest scores alone.

## Data Availability

The original contributions presented in the study are included in the article/supplementary material, further inquiries can be directed to the corresponding author/s.
